# Research in child and adolescent mental Health – How does it relate to clinical research and practice in child and adolescent psychiatry and psychotherapy?

**DOI:** 10.1007/s00787-025-02919-y

**Published:** 2025-11-18

**Authors:** Christine M. Freitag

**Affiliations:** Department of Child and Adolescent Psychiatry, Psychosomatic Medicine and Psychotherapy, University Hospital Frankfurt Goethe Universität, Deutschordenstraße 50, 60528 Frankfurt am Main, Germany

Journals focusing on mental disorders in children and youth receive and accept many articles related to general aspects of child and adolescent mental health. The current issue of ECAP similarly contains more than 50% of articles focusing on symptoms of mental health problems, which may occur in children and youth with manifest mental disorders, but which also are also frequently found in children and youth without a mental disorder. Thus, it is important to explicitly engage in a scientifically based cross-talk between mental health research and clinical research on mental disorders in children and youth, with respect to the translational value of both approaches.

Mental health research focusses on mental wellbeing and associated quality of life. It addresses policies related to prevention and implementation of prevention methods for the general population or a population at a specific risk for deterioration of mental wellbeing (indicated prevention). In addition, service delivery and the impact of policies on wellbeing at the population level (as opposed to the individual level) is addressed by mental health research. As mental health research often targets the population level, it is usually based on large scale studies with easy-to-implement measures. Brief rating scales for parents, teachers, or children and youth above a certain age are often used to study different aspects of mental health.

Clinical child and adolescent psychiatric research on the other hand has its focus on valid and reliable diagnoses of mental disorders, brain (function)-related mechanisms underlying these disorders and their longitudinal course, as well as effective treatments, such as medication, psychotherapy, or brain stimulation, for defined mental disorders in children and youth. Clinical research often uses highly sophisticated study designs, especially within studies on pathophysiological mechanisms, in combination with more detailed clinical rating scales. Regarding symptoms and symptom dimensions derived from rating scales, it is obvious that children and youth with mental disorders will clearly show more symptoms than the population average. These symptoms must have led to current impairment. A combination of a minimum of specific symptoms as well as the presence of specific impairments associated with these symptoms over a longer time-period are the pillars of the current diagnostic criteria of the International Classification of Diseases (ICD) by the World Health Organization or the Diagnostic and Statistical Manual of Mental Disorders (DSM) by the US-American Psychiatric Association.

Results of studies on single symptoms or symptom pattern from population-based or internet-derived selected samples, which were studied by Cénat et al., Farooq et al., Gao et al., Lai et al., Lu et al., Radunz et al., Ke et al., Russel et al. Shalev et al., Yang et al., Wang et al., Wilson et al., Zang et al., Zoellner et al. in this issue, may not necessarily be informative for evidence-based clinical care of children and youth with mental disorders [1]. Such rating scale-based studies may be relevant to gain information on the planning of population-based prevention efforts, albeit with the following caveats:

1. Outcomes need to be clinically relevant and thus should include measures of impairment and duration besides mental disorder derived symptoms to reflect the criteria of a clinical diagnosis. Especially measures of impairment are usually not included in many population-based, longitudinal studies. Alternatively, valid clinical diagnoses can be studied as outcomes (Miron Porto et al., in this issue). Participatory research also has emphasized, that empowerment focused measures are strongly relevant for children and youth. These measures are also rarely included in many population-based, longitudinal studies, even if they focus on the determinants of mental health.
2. Only studies on representative population-based samples, controlled for any relevant risk of bias and with sufficient ethical, data protection and data quality control (such as the ALSPAC [Farooq et al., in this issue], the GUS [Wilson et al., in this issue] or the Millennium Cohort [Russell et al., in this issue]) should be taken into account when discussing (indicated) prevention within a specific health care or social welfare system.
3. Results from internet-based samples (such as Wang et al., in this issue) should not be used to discuss (indicated) prevention, because internet-based samples are most often not representative, show different kinds of biases (selection, coverage, non-response), and an increased measurement error compared to representative samples. Thus, results of such studies cannot be generalized even to the population from which the data were obtained.
4. Results based on population studies only have relevance for countries that are comparable with regard to relevant aspects related to mental health, which are often not directly measurable within a population-based study (such as availability of education, access to health-care and social welfare, environmental legislation and protection, general wealth, access to valid health care information). Otherwise, the risk of false positive or false negative findings due to specific unmeasured, but not randomly distributed risk factor co-aggregations is increased, and irrelevant risk or protective factors may be targeted by prevention interventions.
5. Most importantly, in addition to a mere description of a cross-sectional co-aggregation or prevalence of symptoms, a methodologically valid longitudinal design is a fundamental prerequisite for taking study results into account when planning indicated prevention. Still, even a longitudinal design is not sufficient, as – similar to cross-sectional correlations – also longitudinal correlations cannot be interpreted to reflect causality. More valid indications of possible causality can be derived from more specific study designs and data analyses, such as model based temporal ordering (Racine et al., Ruiz Rivera et al., in this issue), mediation analyses of intermediate processes (Wilson et al., Wang et al., in this issue), or the study of sensitive-period effects and developmental cascades (Gao et al., in this issue).
6. To test efficacy (i.e., the extent to which an intervention does more good than harm when delivered under optimal conditions) or effectiveness (i.e., intervention effects when delivered in real-world conditions) of (indicated) prevention interventions, similar to clinical trials (such as Keuppens et al., in this issue), high-quality randomized controlled trials are of utmost importance, but are less frequently done in mental health research than in clinical research on mental disorders in children and youth [2, 3].

Another critical point related to rating scale-based approaches is the definition of symptoms. In the current issue, five articles carry the term “psychotic” in their title. Of these, one explicitly refers to the diagnostic criteria of a psychotic disorder according to ICD-10 (F23: acute and transient psychotic disorder [Cirnigliaro et al., in this issue]), one implies a clinical diagnosis of ICD-10, chapter F2 as exclusion criterion in the methods section, but does not explicitly refer to ICD or DSM criteria of psychosis (Radha Krishnan et al., in this issue). Two articles refer to “psychotic experiences” (Wang, Wang et al.; Wang, Xu et al., in this issue), and one to “psychotic vulnerability” (Borrelli et al., in this issue). In addition to the phenotypic heterogeneity of psychotic disorders as described in ICD-10, chapter F2, these additional terms of “psychotic experiences” and “psychotic vulnerability” may increase confusion about the concept of “psychosis”. Even the term “psychotic experiences” is differently defined in both articles using this term (Wang, Wang et al.; Wang, Xu et al., in this issue). Wang, Wang et al. (in this issue) are not using an overall definition of “psychotic experiences” in their methods section, but add additional analyses on “hallucinations” and “delusions”. They cite many articles reporting data on the Youth Self Report (YSR), which contains a subscale “Thought problems”. Wang, Xu et al. (in this issue) define “psychotic experiences” according to the 8-item Positive Subscale of the Community Assessment of Psychic Experiences (CAPE-P8), which has been validated in China based on a European 42-item questionnaire for adults (CAPE). The CAPE-P8 assesses two dimensions, hallucinatory experiences (6 items) and delusional experiences (2 items). The term “psychotic vulnerability” (Borrelli et al., in this issue) again is based on the “Thought Problems” subscale, in this article derived from the parent-rated Child Behavior Checklist (CBCL), and not from the self-reported YSR, although the underlying construct of both “Though problems” scales is similar. People from the field of child and adolescent psychiatry and psychotherapy, who are familiar with the CBCL and YRS (https://aseba.org/), are well aware that the “Thought Problems” subscales contain several items which are capturing obsessive-compulsive disorder-related as well as rather unspecific symptoms, and only two items possibly related to visual and auditory hallucinations. In addition, the “Thought Problems” subscale of both instruments shows the lowest internal consistency of all CBCL or YSR-derived subscales (https://aseba.org/wp-content/uploads/2019/01/ASEBA-Reliability-and-Validity-School-Age.pdf). It has been tested for its predictive value to effectively screen for the presence of a high-risk status for psychosis, with insufficient diagnostic accuracy (receiver operating curves < 80%) in two independent studies form Europe [4] and the US [5]. Generally, „psychotic symptoms“ as defined in the studies of this issue, are far more common in the general population of children and youth at a given timepoint (>25%) than the diagnosis of a psychotic disorder according to ICD-10, chapter F2 (~ 0.5%). “Psychotic symptoms” are thus more general indicators of the severity of the underlying mental disorder and are a risk factor for additional depressive and anxiety symptoms (see Borelli et al., in this issue), but not specific for the prediction of a psychotic disorder [6].

Taken together, to align mental health research and clinical research on mental disorders in children and youth with respect to the translational value of both approaches, the symptom-based approach should be complemented by “impairment” and “duration” information to increase its translational clinical relevance, but also by latest information of participatory research with children and youth with mental disorders. In addition, similar definitions and concepts of psychopathological symptoms should be studied across both approaches. Here, the HiTOP approach, i.e. the Hierarchical Taxonomy of Psychopathology, may be helpful to guide future studies [7].
