# Correction to: Research in child and adolescent mental Health – How does it relate to clinical research and practice in child and adolescent psychiatry and psychotherapy?

**DOI:** 10.1007/s00787-026-02970-3

**Published:** 2026-01-20

**Authors:** Christine M. Freitag, Frankfurt Professorin

**Affiliations:** Department of Child and Adolescent Psychiatry, Psychosomatic Medicine and Psychotherapy, University Hospital Frankfurt Goethe Universität, Deutschordenstraße 50, 60528 Frankfurt am Main, Germany

**Correction to: European Child & Adolescent Psychiatry (2025) 34:3327-3329**

10.1007/s00787-025-02919-y

In the Original version of this article, the authors’ names were incorrectly written as “Christine M. Freitag. Frankfurt Professorin” but should have been “Christine M. Freitag”.

The original article has been corrected.

